# Victims' Time Discounting 2.5 Years after the Wenchuan Earthquake: An ERP Study

**DOI:** 10.1371/journal.pone.0040316

**Published:** 2012-07-05

**Authors:** Jin-Zhen Li, Dan-Yang Gui, Chun-Liang Feng, Wen-Zhong Wang, Bo-Qi Du, Tian Gan, Yue-Jia Luo

**Affiliations:** 1 State Key Laboratory of Cognitive Neuroscience and Learning, Beijing Normal University, Beijing, China; 2 Institute of Psychology, Chinese Academy of Sciences, Beijing, China; University of Minnesota, United States of America

## Abstract

**Background:**

Time discounting refers to the fact that the subjective value of a reward decreases as the delay until its occurrence increases. The present study investigated how time discounting has been affected in survivors of the magnitude-8.0 Wenchuan earthquake that occurred in China in 2008.

**Methodology:**

Nineteen earthquake survivors and 22 controls, all school teachers, participated in the study. Event-related brain potentials (ERPs) for time discounting tasks involving gains and losses were acquired in both the victims and controls.

**Findings:**

The behavioral data replicated our previous findings that delayed gains were discounted more steeply after a disaster. ERP results revealed that the P200 and P300 amplitudes were increased in earthquake survivors. There was a significant group (earthquake *vs*. non- earthquake) × task (gain *vs*. loss) interaction for the N300 amplitude, with a marginally significantly reduced N300 for gain tasks in the experimental group, which may suggest a deficiency in inhibitory control for gains among victims.

**Conclusions:**

The results suggest that post-disaster decisions might involve more emotional (System 1) and less rational thinking (System 2) in terms of a dual-process model of decision making. The implications for post-disaster intervention and management are also discussed.

## Introduction

Disasters have a large impact on cognition and decision-making processes [1]–[7], which has led to an ongoing debate regarding whether people become more rational or irrational after a disaster [2]–[5], [8]. For example, after the 9/11 terrorist attacks Americans avoided flying due to a perceived dreaded risk and instead chose to drive, which resulted in more car accidents than usual [3]. As flying is generally considered safer than driving [9], Gigerenzer [3] argued that the avoidance of flying indicated a more irrational tendency after disasters. By contrast, Sacco et al. [2] reported that the events of 9/11 caused outcomes of the decision making process to move closer to those expected from rational choices. Most of the existing studies regarding the effects of disasters on decision making, however, have focused predominantly on risky decision making (i.e., choices involving probabilities) [2], [4], [7]. Few studies have investigated the influence of disasters on decision-making rationality from the perspective of intertemporal choices, despite the fact that intertemporal choices play just as important a role as risky choices in the field of decision making.

Intertemporal choices – decisions involving tradeoffs among outcomes occurring at different points in time [10] – have a major bearing on many everyday life problems, such as decisions about savings, investments, health, education, and even policy debates about long-term challenges (e.g., global warming) [11]–[13]. The typical example of intertemporal choices involves a choice between a smaller, more immediate reward and a larger, more delayed reward [14]. A substantial amount of research shows that people have some form of time preference, with a tendency to prefer immediate rewards rather than delayed ones and, conversely, to prefer losses occurring later over now [10], [13], [15]. Such preferences could be viewed from the perspective of discounting, which assumes that the subjective value of a reward decreases as the delay until its occurrence increases [16]–[18]. Time discounting was found a robust phenomenon that can be observed even in rats and pigeons [19], [20]. Most previous studies have focused on intertemporal choices of gains, with only a few studies on losses. Although discounting of delayed gains and losses could be described by similar discounting functions [14], [21], empirical evidence has suggested that losses and gains are discounted asymmetrically [17], [22]–[24].

To our knowledge, up till now, only quite a few studies have investigated the effect of disasters on intertemporal choices. By studying the same participants before and after the 2008 Wenchuan earthquake, our previous study [25] demonstrated how a major disaster could influence the discounting of delayed gains and losses. The results indicated that, when facing the choice between a sooner and smaller (SS) gain/loss and a later and larger (LL) one, more people after the earthquake compared with before tended to prefer the SS gain and LL loss, but the effect in the loss domain failed to reach statistical significance. However, it is known that field studies are subject to additional confounds due to the inability to control some important factors. There are two such issues in the original work of Li et al. [25]. First, because the earthquake could not be foreseen there was no comparable control group. Hence the differences found in intertemporal choices between the pre-test (which was administered six months before the earthquake) and the post-test (which was administered one week after the event) by Li et al. [25] might be not due to the earthquake per se, but simply to changes over time, even though some previous work has suggested a test-retest reliability for time discounting over a 3-month interval [26]. The second issue with Li et al.'s [25] study is that the number of scenarios used was quite limited, which calls into question the reliability of the findings. Given these limitations, our previous findings [25] need to be further tested.

Time discounting is considered as a bias deviate from rational choices [27]–[29]. Thus, our previous finding [25] that delayed gains were discounted more steeply after a disaster may suggest a less rational tendency in post-disaster decision making if the effect could be verified.

Furthermore, researchers have recently proposed that there are two distinct systems underlying the decision-making process [30]–[34]. System 1 (the intuitive system) is also referred to as ‘affective heuristics’, which is relatively emotional, associative, automatic, contextualized and implicit [34]–[36]; while System 2 (the rational system) is deliberate, rule-based, controlled, and explicit [30], [35], [36]. Judgment and decision making may be based on either or both of these two systems [35]. Choices employed System 2 are essentially rational, whereas decision biases commonly occur in System 1. Neuroscientists have put forth further proposals regarding the brain systems that may support the dual-process model [37]. For example, using functional magnetic resonance imaging (fMRI), McClure et al. [38] hypothesized and tested that the differences between choices made with immediate versus delayed rewards are due to the operation of the two systems in the brain: an emotional, intuitional system (System 1) versus a deliberate, rational system (System 2). Their results show a nice deferential activation of emotional and rational regions for immediate versus delayed reward choices. Specifically, parts of the limbic system associated with the midbrain dopamine system, including the paralimbic cortex, are preferentially activated by decisions involving immediate rewards. By contrast, regions of the lateral prefrontal cortex and posterior parietal cortex are engaged uniformly by delayed gains. Following the call of Breiter *et* *al*. [39] to study the two systems by "using new techniques in neuroscience to provide time-ordered data localized to particular brain areas”, in the current study, we attempt to use event-related brain potential (ERP) methods to investigate how a catastrophic disaster could influence the operation of the two brain systems during the intertemporal decision-making process.

On May 12, 2008, an earthquake measuring 8.0 on the Richter scale shook Wenchuan, China's Sichuan Province, leaving 69,227 people dead, more than 374,000 injured, and approximately 5 million people homeless (as of 25 September, 2008). Based on the finding that victims are still influenced by a major earthquake for two or more years after the event [40]–[43], in the present study, we compared the ERP data recorded during time discounting tasks performed by the disaster-exposed group with those performed by the unexposed population approximately 2.5 years after the Wenchuan Earthquake. There were two main purposes of this study: the first was to test whether the original effect found by Li et al. [25] was due to the disaster rather than time, and the second and primary purpose of this study was to investigate the neural basis of the effect of disasters on intertemporal choices using ERPs, with the hopes of finding neural evidence to contribute to the post-disaster rationality debate.

## Methods

### Ethics

The study was approved by the ethics committee of Beijing Normal University and informed consent was obtained from all participants. A written agreement was signed during the recruitment procedure and all participants verbally reconfirmed their consent to participate before the experiment. Specifically, with the assistance of a local teacher, two members of the study team went door-to-door in the selected school to recruit participants. Each teacher was presented with a one-page recruitment sheet that illustrated the ERP study, informing them of the goals, procedures, study criteria, potential risks (including discomfort), research sites and payment, etc. In addition to the verbal description, there was also a picture of a girl wearing an ERP cap on the recruitment sheet to illustrate the ERP method more clearly. After being fully informed about the study, teachers who met the study criteria and agreed to volunteer were asked to write down their names and contact numbers on the recruitment sheet. In addition to the prior signed agreement on the recruitment sheet, all participants verbally reconfirmed their consent to participate before the experiment began.

### Participants

The selected experimental site was the Bayi Middle School, which is located in the town of Yinghua in the rural city of Shifang, Sichuan Province. This school was selected because it was a rebuilt school that accommodated teachers and students from four schools that were severely damaged by the Wenchuan earthquake. There were more than 200 students and approximately 18 teachers died in the tremendous earthquake, with even more injured. A comparable rural middle school located in a town out of the earthquake area was selected as the control school.

The participants in the experimental group were 19 teachers from Bayi Middle School who experienced the major earthquake but were not injured. The participants in the control group were 22 teachers recruited from the control school who did not experience the earthquake.

The two towns are comparable in their natural/social economic environment; both towns are located at the foot of a hill, and the living/consumer price levels are approximately the same for the two towns. Although there is, to our knowledge, no current data on the Consumer Price Index for the two towns, the descriptions of prices for goods and services when the experiment took place in 2010 may be helpful to illustrate the living/consumer price levels. For example, in both towns the price for a haircut was about ¥20 and for a carwash was about ¥10. The prices of meat, vegetables and fruits were also similar. Financial officials in the two selected schools reported that almost all the teachers earn a monthly salary of ¥3,000 to ¥4,000. There were no significant differences in demographic features between the experimental and control groups (see Table 1).

**Table 1:** Study Sample Demographics.

Area		Earthquake Devastated(N = 19)	Non-devastated(N = 22)
Occupation		Middle School teacher	Middle School teacher
Monthly salary range		¥ 3,000– ¥ 4,000	¥ 3,000– ¥ 4,000
Gender	Male	13 (68.5%)	14 (64.4%)
	Female	6 (31.5%)	8 (36.6%)
Age	Mean	35.16	34.09
	SD	6.26	8.46

All participants were healthy and right handed with normal or corrected-to-normal visual acuity. They were paid for their participation after the experiment. The study was performed from September to October 2010, approximately 2.5 years (29 months) after the earthquake.

### Procedures

The participants performed the experimental tasks in quiet hotel rooms adjacent to the schools.

**Figure 1:**
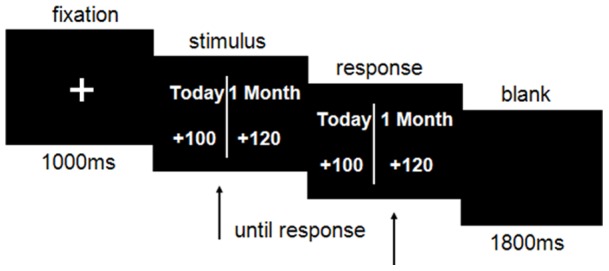
Sequence of events in a single trial of gain task. The loss trials were identical to the gain trials except that there was a “−” sign before the monetary amounts.

The experimental tasks were similar to those employed by former studies [38], [44]. Participants were instructed to choose between two monetary gain/loss (SS *vs*. LL) alternatives at different times (today *vs*. a month later or 1.5 months later) (See Figure 1). For each set of the intertemporal alternatives, the early dollar amount was randomly drawn from a Gaussian distribution with a mean of ¥100 and standard deviation of ¥50. The percentage difference between the larger and the smaller alternatives was selected from the set {5%, 10%, 15%, 25%, 35%, 50%}. In total there were 16 practice trials and 256 test trials, with an equal number of gain and loss trials. The intertemporal choices of gains and losses were identical except the signs (“+” or “−”) before the monetary amount, indicating that money would be gained or lost at the corresponding time. The choices were presented in a pseudo-random order.

The two alternatives for each choice were presented on either side of the screen. Participants were instructed to press the “F” or “J” key on the computer keyboard to indicate their choice.

### EEG recording

The electroencephalogram (EEG) was recorded from 64 scalp sites using electrodes mounted on an elastic cap (Brain Product, GmbH, Germany), with an online reference to the left mastoid. The horizontal electroencephalogram (HEOG) was recorded with two electrodes placed laterally to the right and left eyes. The vertical electroencephalograms (VEOG) were recorded with electrodes placed above and below the right eye. All inter-electrode impedances were maintained below 10 kΩ. The EEG and EOG were amplified using a 0.01–100 Hz band-pass and continuously sampled at 500 Hz in each channel for off-line analysis. All EEG signals were re-referenced off-line to the average of the left and right mastoids. The EEG data were low-pass filtered below 30 Hz (24 dB/oct) and were corrected for eye movements and/or blinks with the Gratton and Coles' method as implemented in the Brain Vision analysis software (Brain Product, GmbH, Germany). Trials containing EEG sweeps with amplitudes exceeding ± 100 μV were excluded.

For each stimulus, epochs of 1000 ms duration including a 100 ms pre-stimulus period were extracted from the continuous EEG record. Because the P200 and P300 components are components commonly found in decision-making processes, we analyzed the P200, P300 and N300 components.

## Results

### Behavioral results

**Figure 2:**
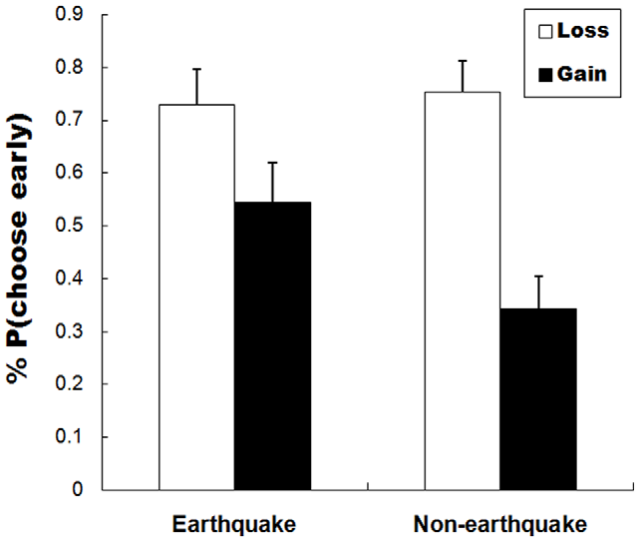
Percentage choices of sooner and smaller (SS) gains/losses in earthquake-devastated and non-devastated areas.

In each trial, participants indicated their preferences for SS or LL by pressing the corresponding key. The percentage of SS gain/loss choices in the earthquake-devastated and non-devastated areas are illustrated in Figure 2.

A chi-square test revealed that the SS options involving gains were chosen more in the earthquake-devastated compared with non-devastated areas (*p*<0.05), suggesting that delayed gains were discounted more by earthquake victims than by the control group. Additionally, fewer trials involving an SS loss were chosen in the earthquake-devastated (72.8%) than in non-devastated (75.4%) area, but this did not reach statistical significance (*p*>0.05).

There was no significant difference in reaction time between groups for both gain and loss tasks (*p*>0.05).

### ERP Results

Repeated measures analyses of variance (ANOVAs) for each ERP component were conducted with group (earthquake vs. non-earthquake) as a between-participants factor, and with task (gain *vs*. loss) as a within-participants factor. Another within-participants factor was the electrode, which included Fz, F3, F4, FCz, FC3 and FC4 for the P200 component; Pz, P3, P4, POz, PO3, PO4, CPz, CP3 and CP4 for the P300 component; and Fz, FCz, C z for the N300 component. These electrode sites were chosen based on the literature [45], [46] and after visual inspection of the ERP grand average waveforms. In all the analyses, the Greenhouse-Geisser correction for non-sphericity was applied where appropriate.

**Figure 3:**
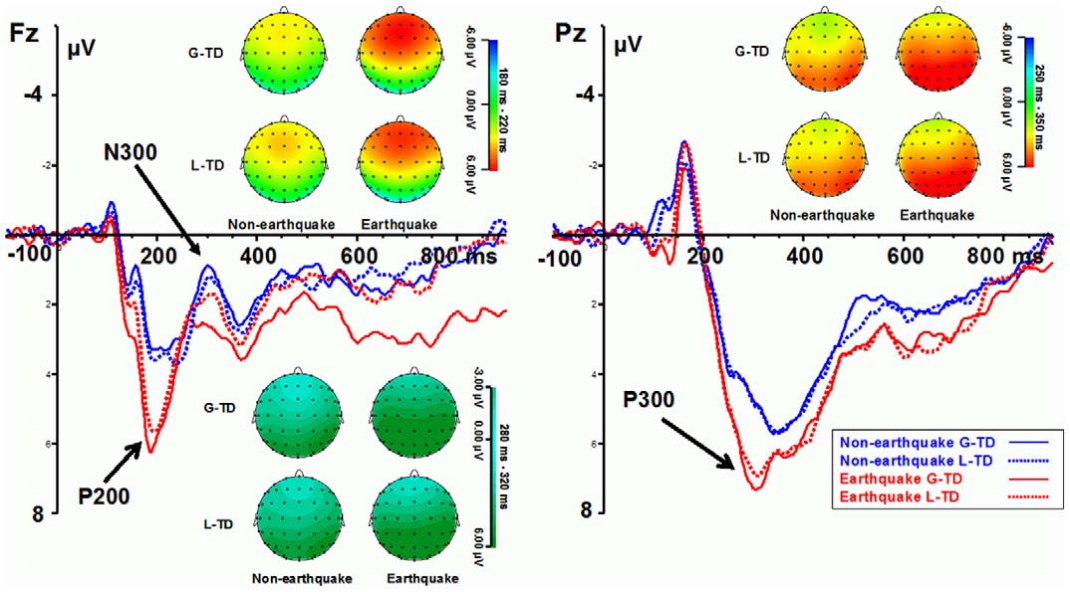
Grand average ERPs and scalp potential maps (at the time range of P200, N300 and P300 components) on time discounting tasks involving gains (G-TD) and losses (L-TD) for the earthquake and non-earthquake groups.

Figure 3 shows the grand average ERPs at the sites of the maximum amplitude (Fz for the P200 and N300, Pz for the P300) as well as scalp potential maps for the earthquake and non-earthquake groups for time discounting tasks involving gains (G-TD) and losses (L-TD).

#### P200

The P200 amplitudes was measured as the average amplitudes of the waveform in a window from 180–220 ms. ANOVAs indicated that the P200 amplitudes differed significantly between the earthquake and non-earthquake groups (F(1, 39)  = 4.471, *p*<.05). The P200 amplitudes for the earthquake-devastated group were larger than those for the non-devastated group. No other effects were significant.

#### P300

The P300 amplitudes were measured as the average amplitudes of the waveform in a window from 250–350 ms following stimulus presentation. P300 amplitudes showed significant main effect of group (F(1, 39)  = 4.288, *p*<.05). The P300 amplitudes for the earthquake-devastated group were larger than those for the non-devastated group. No other effects were significant.

#### N300

The N300 amplitude was calculated as the average amplitudes of the waveform in a window from 280–320 ms following stimulus presentation. For the N300 amplitude, a significant interaction was found between group and task (F(1, 39)  = 6.562, *p*<.05). Further simple effects analyses revealed that, for gain tasks, the N300 amplitude was marginally significant smaller in the earthquake-devastated group compared with the control group (*p* = 0.059). With regard to the N300 amplitude for loss tasks, there was no significant difference found between groups (*p* = 0.555). In addition, the N300 amplitude for gains and losses showed a marginally significant difference (*p*s<.1) in both the earthquake and non-earthquake groups.

Repeated ANOVAs for the P200, P300 and N300 were also conducted with group (earthquake vs. non-earthquake) as a between-participant factor, and with choice (sooner vs. later) and electrode as within-participant factors. The Greenhouse-Geisser correction for non-sphericity was applied when necessary. Only participants with at least 20 trials in either condition (sooner and later) were taken into account. Finally, there were 20 participants (7 in the experimental group and 13 in the control group) included in the analysis. Only the results for gains are presented in detail because there were insufficient data (too few participants) for losses to allow for a meaningful ERP analysis.

ANOVAs revealed that neither the effect of group (P200: F(1, 18)  = 0.03, *p*>.05; P300, F(1, 18)  = 0.530, *p*>.05; N300: F(1, 18)  = 0.008, *p*>.05) nor the effect of choice (P200: F(1, 18)  = 0.17, *p*>.05; P300, F(1, 18)  = 1.432, *p*>.05; N300: F(1, 18)  = 2.4, *p*>.05) were significant. It is worth noting that the group effect was significant in the group (earthquake vs. non-quake) × domain (gain vs. loss) × electrode ANOVA. Therefore, the insignificance of choice effect might be due to one of these two main factors: (1) insufficient number of participants and (2) imbalance number of epochs between the sooner and the later choices, which is likely to influence the amplitude of the averaged ERP.

### Source Analysis

Source analysis was performed for components that differed between the groups using standardized low-resolution brain electromagnetic tomography (sLORETA) [47], which is a tomographic technique that gives a solution to the so-called inverse problem (i.e., the computation of images of electric neuronal activity based on extracranial measurements). sLORETA yields images of standardized current density with zero localization errors, allowing a more precise source localization than the older LORETA method [48]. It has been previously shown that sLORETA can achieve a reliable localization of possible underlying sources [49], [50].

**Figure 4:**
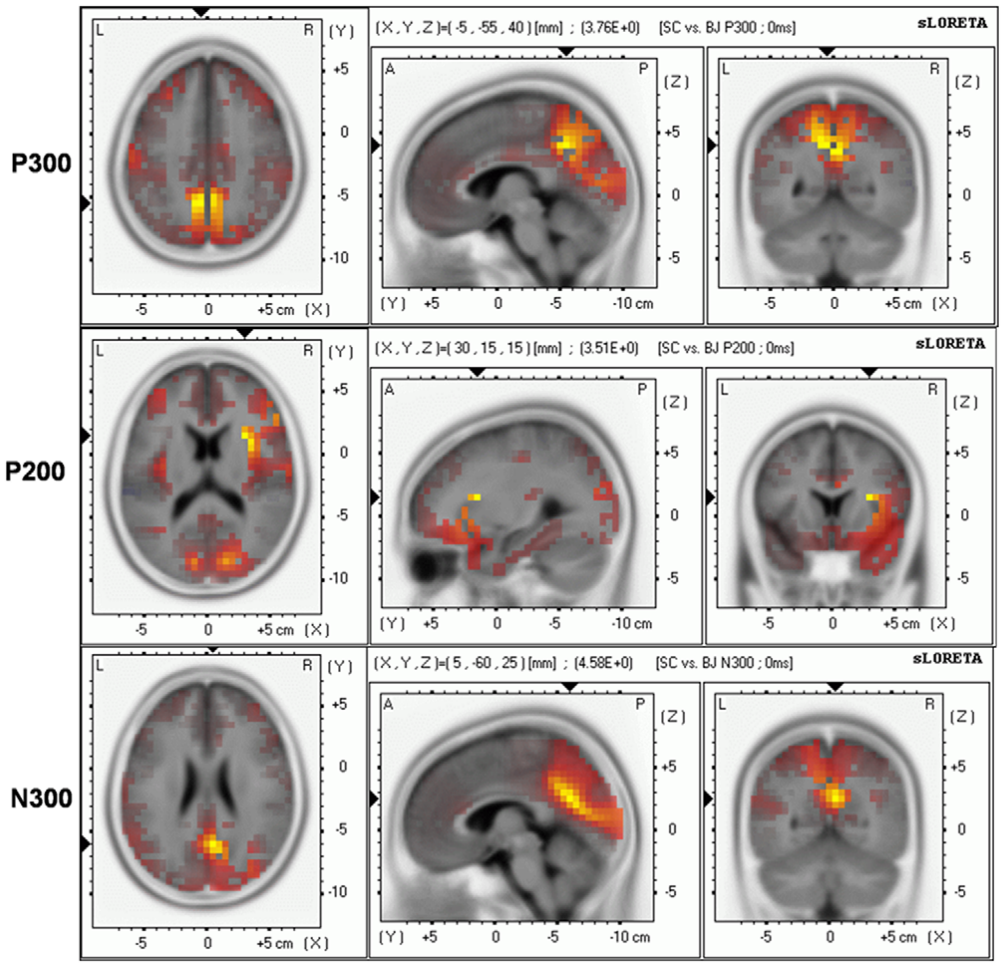
Graphical representation of the sLORETA results comparing the ERPs between the earthquake and control groups at the time range of P300, P200 and N300. Orange indicates significant hyperactivity for earthquake survivors compared with controls.

As shown in Figure 4, significant sources relating to the difference in the time range of the P200 component between the experimental and control groups are located in the insula (BA 13) and inferior frontal gyrus (IFG; BA 44, BA 45). For the N300 and P300 components the difference is mainly located in the cingulate gyrus (BA 31) and precuneus (BA 7, BA 23). In all of these regions the earthquake group showed increased activity compared with controls.

## Discussion

In the previous study [25] conducted shortly after the Wenchuan Earthquake, we found that delayed gains were discounted more steeply after compared with before the earthquake, while the effect in the loss domain did not reach statistical significance. One of the biggest drawbacks of our prior work [25] is the lack of a comparable control group, which raised the question that the reported effect might be not due to the disaster. By having teachers from earthquake-devastated and non-devastated areas as participants, this study re-examined the influence of the Wenchuan Earthquake on victims' time discounting. The behavioral results from the current study are consistent with those of our prior work [25], suggesting that the effect was caused by the disaster and remained robust even two more years passed by. In addition, it has been repeatedly found that people's discounting of delayed gains is influenced more by the earthquake than delayed losses, which can be interpreted, as explained by Li et al. [25], as due to the asymmetry in discounting processes of future gains and losses [22], [23], [44], [51].

The ERP data were of particular importance in specifying the influence of the earthquake on intertemporal decision making. The time discounting task elicited larger P200 and P300 amplitudes in earthquake survivors than in the control group. Furthermore, there was an interaction for the N300 amplitude between group (earthquake *vs*. control) and task (gain *vs*. loss).

### P200

It is assumed that the P200 is related to early stimulus encoding, reflecting stimulus detection, semantic processing and the early stages of decision making. Recently, Polezzi et al. [52] reported that unpredictable outcomes elicited larger P200 amplitudes than did predictable outcomes. The increased P200 amplitudes among earthquake victims may reflect that, after a disaster, future events seem more unpredictable.

### P300

The P300 is widely reported as a component linked to risky decision making [53]–[55]. The P300 amplitude is found to vary with event probability and outcome magnitude [53], [56]. Larger amounts and lower probabilities elicit a larger P300 [53], [56]. P300 amplitude was also found to reflect risk-taking behavior, with larger amplitudes in the context in which participants showed a higher risk-tendency [56]. Generally, the P300 seems to reflect characteristics of the evaluation process for making a decision, with larger P300 amplitudes being associated with more risky alternatives or behaviors.

One explanation for intertemporal discounting is that throughout evolutionary history future rewards have been uncertain [57]; time delays are associated with implicit risks [58]. With longer delays there could be a greater risk that the expected or promised reward will not actually be received [57], [59]. Thus, delayed positive consequences are avoided because they are less certain (time introduces a risk component) [59]. From this perspective, the enhanced P300 found among the survivors could reflect that time delays were viewed as even more risky after the earthquake. According to Tversky and Kahneman's [60] availability and representativeness heuristics, a catastrophic disaster may make the uncertainty of receiving a later gain more salient because an intervening event might block the possibility of making it real.

### N300

Time discounting is often used as a measure of compulsivity or self- control [61], [62]. A higher discounting rate indicates a higher focus on immediate compared with delayed events, i.e., more impulsive and less controlled. Within the field of cognitive neuroscience, impulsivity is often equated with the term ‘disinhibition’, referring to the idea that top-down control mechanisms ordinarily suppress automatic or reward-driven responses that are not appropriate to the current demands [63]. A large amount of literature has emerged on the role of the anterior N2 component, a negative wave peaking between 200 and 350 ms after stimulus onset, in inhibitory control process [45], [64], [65]. For example, various studies using go/no-go tasks have found that larger N2s are elicited by “no-go” trials than “go” trials [45], [64], [65], suggesting that the N2 component is consistently linked to inhibitory control. Therefore, it is appropriate to identify the N300 found in the present study – peaking within 200 and 350 ms with an anterior scalp distribution – as an N2 component, reflecting the process of inhibiting the impulsivity towards the immediate rewards rather than delayed larger ones. The statistical analysis revealed that the N300 amplitude for gain tasks tended to decrease in the experimental group, suggesting that there might be a deficiency in inhibitory control for gains among earthquake survivors. This group difference was not true for loss tasks. This pattern of results may provide a further neural explanation for the repeated behavioral findings that discounting of delayed gains was more influenced by the earthquake than delayed losses.

The sLORETA analysis of the ERP components that differed between the groups (the P200, P300 and N300) revealed significant hyperactivity for the earthquake group in the insula (BA 13), cingulate gyrus (BA 31), precuneus (BA 7, BA 23) and inferior frontal gyrus (BA 44, BA 45). This pattern of findings indicates more activation of emotion-related areas including the insula and cingulate gyrus [66], [67] in the earthquake group, suggesting that earthquake survivors employed a more emotional, intuitional system to make their decisions. According to McClure et al.'s [38] findings that the activation of emotional brain areas predicted choices for immediate rewards, the hyperactivity of emotion-related regions among victims were congruent with our behavioral findings that immediate gains are chosen more by earthquake survivors than controls. Notably, it has been proposed that the activation of the precuneus is involved in source memory (in which the “source” circumstances of a memory are recalled), especially during the regeneration of previous episodic contextual associations [68]–[70]. In line with Tversky and Kahneman's [71] ‘availability heuristic’ of intuitive decision making, that individuals estimate the likelihood of an event “by the ease with which instances or associations come to mind”, the hyperactivity of the precuneus among earthquake survivors suggests that victims referred to their past earthquake experience when making decisions involving delayed gains and losses. In a recent fMRI study [72] it was found that the IFG has an inhibitory and risk-averse role in decision making, with increasing IFG activity to higher risk aversion. Furthermore, according to Christopoulos et al. [72], the IFG “does not influence the objective evaluation of risk but rather the subjective perception of the riskiness of the option”. The increased activations of the IFG in the earthquake group suggest that other options with the same time delay were perceived to be more risky and, therefore, resulted in higher risk aversion (i.e., more selection of the immediate gains). It is worth mentioning that, the sLORETA analysis provides only an estimate of the source(s) for the various ERP components, since sLORETA inherently would not produce a pinpoint solution for a point source [47], and its present implementation offers only a spatial resolution of 5 mm. Therefore, the source analysis results presented here are suggestive, not definitive, evidence of specific brain areas being affected by the earthquake. Further research using neuroimaging methods, such as fMRI, is needed to specify the brain regions associated with post-disaster decision making.

Our results provided convergent evidence that post-disaster decisions may be less rational, involving more emotional thinking (System 1) and less rational thinking (System 2) in terms of a dual-process model of decision making. First, the behavioral findings that the immediate gains were chosen more by the earthquake survivors than by the control group implies a more irrational tendency among victims, because discounting future gains is viewed as an irrational decision bias which “ought to be resisted” [29]. Second, the ERP results suggest less inhibitory control (i.e., less rational decision making) for immediate gains after the earthquake than in normal circumstances, which electrophysiologically implies a less rational/cognitive tendency after the disaster as well. Third, the sLORETA analysis indicates more activation of emotion-related regions among earthquake survivors. From an evolutionary standpoint [35], [73], [74], in the environment of human ancestors, most decisions relied on the operation of System 1, with System 2 only needed to deal with some unusual cases. In modern society, however, the more sophisticated environment may require more and more System 2 thinking. The results from the current study imply that, after a disaster, people seemed to make irrational regressions towards the thinking style of our ancestors, with a heavier reliance on System 1 when making a decision.

One limitation of the present study is the relatively small number of participants included in the ERP analysis distinguishing between immediate and delayed choices, which may lead to the argument that the statistical approach was not sufficiently robust to produce clearly significant results. Further research is needed to clarify this issue.

The findings of the present study highlight the lasting influence of the Wenchuan earthquake and its neural mechanism. The results indicate that immediate gains were more preferred by the earthquake survivors than by people who did not experience the earthquake. It might be inferred from the results that a major natural disaster could cause people to have less inhibitory control and to evaluate future gains as more risky, leading to more shortsighted decisions involving intertemporal tradeoffs. The current data behaviorally and electrophysiologically suggest that post-disaster decisions may be less rational. As mentioned above, time discounting plays an important role in many everyday life problems, such as decisions about investments and savings, dieting, education, marriage, alcohol and drug use/abuse, etc. The current findings can then also be related to theoretical and policy implications for a better understanding of post-disaster behavior and for effective intervention and management in the aftermath of a disaster.
